# Editorial Note: Construction of a high-density genetic map and QTL analysis for yield, yield components and agronomic traits in chickpea (*Cicer arietinum* L.)

**DOI:** 10.1371/journal.pone.0355892

**Published:** 2026-08-12

**Authors:** The PLOS One Editors

Following publication, this article [1,2] was reassessed by a member of the *PLOS One* Editorial Board who was not involved in the original peer review of [1]. Based on their input, the journal concluded that the article meets the journal’s core criteria, although there are some reporting issues. Specifically:

- The title does not appear to be fully supported given that the chromosome maps in Fig 1 are of varying density.
- There appears to be a low correlation between years of the agronomic study.
- Statements on molecular mechanisms and possible functions of some genes in the Discussion section appear vague.
- There appears to be an error in the Materials and methods section: the area of a 4 x 3 m plot should be listed as 12 m^2^ rather than 1.2 m^2^.

With this notice, PLOS considers the concerns in [2] resolved.

## References

[1] Barmukh R, Soren KR, Madugula P, Gangwar P, Shanmugavadivel PS, Bharadwaj C, et al. Construction of a high-density genetic map and QTL analysis for yield, yield components and agronomic traits in chickpea (*Cicer arietinum* L.). PLoS One. 2021;16(5):e0251669. doi: 10.1371/journal.pone.0251669 33989359 PMC8121343

[2] The *PLOS One* Editors. Expression of Concern: Construction of a high-density genetic map and QTL analysis for yield, yield components and agronomic traits in chickpea (*Cicer arietinum* L.). PLoS One. 2026;21(6):e0350350. doi: 10.1371/journal.pone.0350350 PMC13225392 42224220

